# Supplementary material for: Exploring the Use of Antibiotics for Dental Patients in a Middle-Income Country: Interviews with Clinicians in Two Ghanaian Hospitals
Source: Antibiotics (Basel). 2022 Aug 9;11(8):1081. doi: 10.3390/antibiotics11081081 (PMC9404843; doi:10.3390/antibiotics11081081)
Supplement: Supplementary file 1 [file antibiotics-11-01081-s001.zip › antibiotics-1859837-supplementary.pdf]

Supplementary information File S1

Participant information sheet

YOU WILL BE GIVEN A COPY OF THIS INFORMATION SHEET

Title of project: Dental Antibiotic Stewardship in Ghana: A scoping study at the Keta Municipal Hospital and the Ghana Police Hospital.

Introduction

We would like to invite you to participate in this research. Before you decide whether you want to take part, it is important for you to understand why the research is being done and what your participation will involve. Please take time to read the following information carefully and discuss it with others if you wish. You can ask me if there is anything that is not clear or if you would like more information.

What is the purpose of the project?

The purpose of this project is to understand the use of antibiotics for dental conditions by specific health care providers in Ghana. The findings will provide evidence on how and why dental staff prescribe antibiotics and the factors which influence their behaviour. We would also like to identify any learning needs of the staff around antibiotic stewardship. This information can be used to make recommendations about standard protocols and best practice in dental care.

Why have I been invited to take part?

You are being invited to participate in this project because you have experience as a dental prescriber in a public hospital in Ghana. We want to understand your experiences in antibiotic prescribing and how this impacts your work and patient outcomes.

What will happen if I take part?

If you choose to take part in the project you will be asked to participate in an interview to help us better understand your work with dental antibiotics. The interview will be conducted over the phone or by using online applications such as Zoom, Skype or Google Meet. The interview will include questions about your experience of prescribing dental antibiotics, your knowledge of dental antibiotic stewardship and your views on what influences dental antibiotic use in Ghana. We will also ask you for suggestions on how to develop best practice guidelines on dental antibiotic prescription processes. The interview will last approximately 30 minutes. With your permission the interview will be recorded. The recorded interviews will be typed up without any identifying information to help us with our analyses.

Do I have to take part?

Participation in this research is completely voluntary. You should only take part if you want to and choosing not to take part will not disadvantage you in any way. You are also free to decide not to answer some questions during the interview. Once you have read the information sheet, please contact us if you have any questions that will help you make a

decision about taking part. If you decide to take part, we will ask you to sign a consent form and you will be given a copy of this consent form to keep.

Incentives

On completion of the interview, we will give you a small token to reimburse you for your time and any data costs for the interview.

What are the possible risks and benefits of taking part?

In this study we are interested in understanding your experiences with prescribing dental antibiotics in your hospital. We do not anticipate that there will be any risks your participation in this study. **Participants should be assured that the information they provide will not be used against them by the hospital management or heads of pharmacy department.** Due to the coronavirus pandemic, we will conduct all interviews remotely in order to ensure that participation in this study does not put you at risk of infection.

The results from this study will provide evidence on the factors which are important to pay attention to, with regard to prescriptions of dental antibiotics. Such information can be useful for making policy recommendations and developing guidelines for practice.

Data handling and confidentiality

Any data that are collected from you will be processed under the terms of UK data protection law (including the UK General Data Protection Regulation (UK GDPR) and the Data Protection Act 2018), as well as the Ghana Data Protection Act 2012. You can choose whether or not you would like us to use your real name when we report the results of this study. If you wish to not be identified, we will give you a pseudonym in any publication or dissemination activities. Whether you use your real name or not, we will remove any details which can be used to identify you in any of our reports on findings. If you wish, we can send you a draft copy of any report or publication in which your real name is used for your approval before it is submitted for publication. If you do not approve, we will remove your name or the sections that apply to you from the report.

Any information obtained from you will be treated as confidential, and will only be shared with the research team. However, when we send the audio recordings to be transcribed it is possible that the audio recordings may contain personal information. In order to protect your privacy, we will ask all people who do the transcribing to sign a confidentiality agreement. Any data collected will be shared using secure file-sharing services (such as OneDrive). We will remove your personal details from the final copies of the transcripts before storage and the transcriber will destroy their copy of the recording and transcript. Any documents containing data will be stored on a password-protected computer and hard copies kept in locked cabinets. All audio recordings and transcripts will be kept for up to a year, after which they will be destroyed.

What if I change my mind about taking part?

Your participation in this study is entirely voluntary and you are free to withdraw at any point of the project, without having to give a reason. Withdrawing from the project will not affect you in any way. Even after the interview has been completed, you will be able to

withdraw your data from the project until data analysis begins. After this withdrawal of your data will no longer be possible because your information will have been analysed and included in the final report. If you choose to withdraw from the project, we will not retain the information you have given thus far.

How is the project being funded?

This project is being funded by the Commonwealth Partnerships for Antimicrobial Stewardship (CwPAMS)

What will happen to the results of the project?

The results of the project will be summarised in a report which will be used to engage other researchers, policy makers and other relevant organisations. **This report will be made available to the two hospitals and the pharmacy leads so that participants can access a copy.** The results of this study will also be published in academic journals and presented at conferences and seminars.

Who can I contact for further information?

If you have any questions or require more information about this project, please contact me using the following contact details:

**Dr Wendy Thompson (UK)**<br>University of Manchester<br>Tel.: +44 (0) 7837 190387<br>Email: wendy.thompson15@nhs.net	**Dr Lily Kpobi (Ghana)**<br>Regional Institute for Population Studies<br>University of Ghana<br>Tel.: +233 (0)50 929 5509<br>Email: lkpobi@ug.edu.gh
**Dr Israel Sefah (KMH)**<br>Specialist pharmacist<br>Keta Municipal Hospital<br>Tel.: +233 (0) 20 916 4151<br>Email: iseffah1980@gmail.com	**Dr Daniel Afriyie (GPH)**<br>Director of Pharmacy<br>Ghana Police Hospital<br>Tel.: +233 (0) 24 446 0235<br>Email: acpdank77@gmail.com

What if I have further questions, or if something goes wrong?

This study has been assessed and approved by the Ghana Health Service Ethics Review Committee. If you have any concerns about the way the study has been conducted, or about your rights as a research participant you can contact the research ethics committee using the details below:

Nana Abena Apatu
Ghana Health Service Ethics Review Committee
Tel.: 00233- 50-353-9896
Email: ethics.research@ghsmail.org

Thank you for reading this information sheet and for considering taking part in this research.

Participant consent form

Please complete this form after you have read the Information Sheet and/or listened to an explanation about the research

<table>
<tr><td colspan="2">Title of project: Antibiotic stewardship in Ghana: A scoping study</td></tr>
<tr><td colspan="2">Ethical review reference number: GHS-ERC 005-05-21

Participant Name & ID:</td></tr>
<tr><td></td><td>Tick or initial</td></tr>
<tr><td>1. I confirm that I have read and understood the information sheet dated ………… for the above project. I have had the opportunity to consider the information and asked questions which have been answered to my satisfaction.</td><td></td></tr>
<tr><td>2. I consent voluntarily to be a participant in this project and understand that I can refuse to take part and can withdraw from the project at any time, without having to give a reason.</td><td></td></tr>
<tr><td>3. I consent to the processing of my personal information for the purposes explained to me in the Information Sheet. I understand that such information will be handled under the terms of UK data protection law, including the UK General Data Protection Regulation (UK GDPR) and the Data Protection Act 2018, as well as the Ghana Data Protection Act 2012.</td><td></td></tr>
<tr><td>4. I understand that confidentiality and anonymity will be maintained, and it will not be possible to identify me in any research outputs unless I request to be identified</td><td></td></tr>
<tr><td>5. I agree to be partially identified (my real name only) in any research outputs, as explained in the Information Sheet</td><td></td></tr>
<tr><td>6. I agree to my data being shared with a third-party transcriber who will have signed a confidentiality agreement.</td><td></td></tr>
<tr><td>7. I consent to my participation in the research being audio recorded.</td><td></td></tr>
<tr><td>8. I understand that the information I have submitted will be published as a report</td><td></td></tr>
<tr><td>9. I wish to receive a copy of the final report. The report can be sent to me at the following email address: ____</td><td></td></tr>
<tr><td>10. I agree to be re-contacted in the future by the researchers regarding this project.</td><td></td></tr>
<tr><td>11. I agree that the researcher may retain my contact details so that I may be contacted in the future by the researchers who would like to invite me to participate in future studies of a similar nature.</td><td></td></tr>
</table>

PARTICIPANTS' STATEMENT

I acknowledge that I have read or have had the purpose and contents of the Participants' Information Sheet read and all questions satisfactorily explained to me in a language I understand (……*name of language*). I fully understand the contents and any potential implications as well as my right to change my mind (i.e. withdraw from the research) even after I have signed this form.

I voluntarily agree to be part of this research.

Name of Participant………………………..

Participants' Signature ……………………..OR Thumb Print…………………………

Date: ……………………………….

STATEMENT OF WITNESS

I was present when the purpose and contents of the Participant Information Sheet was read and explained satisfactorily to the participant in the language he/she understood (….*name of language*)

I confirm that he/she was given the opportunity to ask questions/seek clarifications and same were duly answered to his/her satisfaction before voluntarily agreeing to be part of the research.

Name: ………………………

Signature………………………….. OR Thumb Print ……...............................

Date: …………………………

INVESTIGATORS' STATEMENT AND SIGNATURE

I certify that the participant has been given ample time to read and learn about the study. All questions and clarifications raised by the participant have been addressed.

Researcher's name…………………………………….

Signature ………………………………………………

Date…………………………………………………….

Supplementary information File S2

Interview guide

Introduction

Hello, my name is _________ (*researcher's name*). I'm a researcher from _______ (*researcher's affiliation*). Thank you for taking part in this study looking at antibiotic use for dental infections in Ghana. As part of the study, we will be talking to doctors, dentists, pharmacists, nurses and other healthcare professionals working hospitals in Keta Municipal Hospital and Ghana Police Hospital. The aim is to gain insight into your experience of prescribing antibiotics for patients with dental infections and other conditions. We are also interested in your knowledge about optimising the use of antibiotics – which is sometimes called antibiotic stewardship. Our call today will be audio-recorded and should take no more than half an hour.

Is that OK? Yes / No

If no – Thank them for their time and ask them if they can tell you why: (END INTERVIEW)

Do you have any questions? If yes – write their response & interviewers response below:

Interview

- Are you happy to proceed with the interview? I will start the recording now.
- Could you give me some background details?
    - What is your professional role? ______________________________
    - When did you get your qualification? __________________________
    - Do you have any specialist qualifications? ________________________

Topic 1 – Experience of prescribing dental antibiotics – most recent experience

First, I'm interested in your experience of using antibiotics when caring for people with dental problems.

- Tell me about the last time you prescribed antibiotics for a dental problem.
  Prompt: What setting? What dental condition/treatment? Patient – adult/child? What drug type? What duration? Any other dental procedure (e.g., extraction) on the same day?

  - What was your goal of prescribing the antibiotic?

  ____

  - How confident are you that it fixed the problem?

____

Topic 2 – Influences on managing dental conditions with antibiotics

- Thinking more generally about your experience, how often do you prescribe antibiotics to people with dental infections? And what sort of dental infections do you commonly treat with antibiotics?
  Prompt: Every day/week? Clinical indication from STG: Necrotising Ulcerative Gingivitis, Ludwig's Angina, Chronic Periodontitis, Odontogenic infection (abscess). Other may include inappropriate conditions such as dental pain called pulpitis or apical periodontitis

- What influences whether you prescribe antibiotics for dental conditions?
  Prompt: Rarely – sometimes – often – every time

- How often do you think that people you treat want antibiotics for their dental conditions?
  Prompt: Rarely – sometimes – often – every time

- For dentists only: In what other clinical situations do you prescribe antibiotics?
  Prompt: Periodontitis (gum disease)? Prophylaxis to prevent infections such following an extraction, following a root canal treatment or for people at risk of infective endocarditis.

Topic 3 - Barriers and facilitators to effective dental antibiotic stewardship.

In this section I'm interested in your knowledge about and experience of ways in which antibiotic prescribing is being reduced as far as is safely possible in order to reduce the development and spread of antibiotic resistance. These approaches are sometimes called antibiotic stewardship.

- Tell me what you know about antibiotic resistance and stewardship
  Prompts – Problem for dentistry?

- Tell me about the guidelines which you follow for dental antibiotic prescribing
  Prompts – Standard Treatment Guidelines? Any issues with them? Role of pharmaceutical representatives

- Have you ever received any training about antibiotic stewardship?
  Prompts – Where? When? By whom?

- What helps you or makes it hard for you to prescribe in accordance with guidelines?
  Prompts – Environmental eg availability of drugs, access to dental procedures, managers

Topic 4 – Views on what else influences dental antibiotic use in Ghana

This is now the last section. It's our opportunity to pick up anything else not already covered. What do you feel you are the key things which influence antibiotic prescribing for dental patients?

Prompt: Availability of drugs? Access to dental procedures?

Is there anything else which occurs to you that might help understanding dental antibiotic prescribing in Ghana?

Thank you very much for sharing your experience with me. I really appreciate you giving me your time and insight. The study should be complete later this year and you will be able to access a summary of the overall results from the lead pharmacist in your hospital.
